# Supplementary material for: A high-resolution mRNA expression time course of embryonic development in zebrafish
Source: eLife. 2017 Nov 16;6:e30860. doi: 10.7554/eLife.30860 (PMC5690287; doi:10.7554/eLife.30860)
Supplement: Supplementary file 6. [file elife-30860-supp6.zip › biolayout-clusters-files/Cluster244-genes.html]

Cluster244


# Cluster244: Genes

| Ensembl ID | Gene Name | Chr | Start | End | Biotype |
| --- | --- | --- | --- | --- | --- |
| ENSDARG00000006420 | atrnl1b | 12 | 29727934 | 29917286 | protein\_coding |
| ENSDARG00000077694 | dtnba | 20 | 47412304 | 47470456 | protein\_coding |
| ENSDARG00000078942 | enox2 | 14 | 22778882 | 23182176 | protein\_coding |
| ENSDARG00000041728 | man1a2 | 9 | 20743311 | 20954461 | protein\_coding |
| ENSDARG00000075853 | sh3kbp1 | 24 | 25649255 | 25758280 | protein\_coding |
| ENSDARG00000030448 | sppl2 | 2 | 37498212 | 37520552 | protein\_coding |
